# The prevalence of stunting among children and adolescents living in the Middle East and North Africa region (MENA): A systematic review and meta-analysis

**DOI:** 10.7189/jogh.11.04070

**Published:** 2021-12-25

**Authors:** Hassan Joulaei, Parisa Keshani, Mahkameh Ashourpour, Peyman Bemani, Sanaz Amiri, Jamileh Rahimi, Mohsen Aliakbarpour, Amin Salehi-Abargouei

**Affiliations:** 1 Shiraz HIV/AIDS Research Center, Institute of Health, Shiraz University of Medical Sciences, Shiraz, Iran; 2 Health Policy Research Center, Institute of Health, Shiraz University of Medical Sciences, Shiraz, Iran; 3 Department of Nutrition Sciences, School of Health, Larestan University of Medical Sciences, Larestan, Iran.Emam Reza Teaching Hospital, Larestan University of Medical Sciences, Larestan, Iran; 4 Department of Immunology, School of Medicine, Isfahan University of Medical Sciences, Isfahan, Iran; 5 Department of Epidemiology, Health School, Shiraz University of Medical Sciences, Shiraz, Iran; 6 Department of Epidemiology and Biostatistics, School of Public Health, North Khorasan University of Medical Sciences, Bojnurd, Iran; 7 Shiraz HIV/AIDS Research Center, Institute of Health, Shiraz University of Medical Sciences, Shiraz, Iran; 8 Nutrition and Food Security Research Center, Department of nutrition, School of Public Health, Shahid Sadoughi University of Medical Sciences, Yazd, Iran

## Abstract

**Background:**

Given the strategic importance of the MENA, the state of war and inequity in the region and its effect on malnutrition which leads to mortality and reduced economic development in this region, the current study purposed to examine the prevalence of stunting as an indicator of chronic malnutrition in the MENA region, with consideration given HDI, rural/urban area, and war-involved countries.

**Methods:**

The electronic databases of PubMed, SCOPUS, Web of science, and Embase were systematically searched, and English-language articles published between January 1, 2009 and December 31, 2019 were included in this study. The POLIS (population, outcome, location, indicator, study design) criteria were used to perform the systematic review, and studies involving children 2 to 18 years of age were selected.

**Results:**

Fifty-eight (n = 2 202 869) were included based on the study's inclusion criteria. The prevalence of stunting in children in the total MENA region was 22.0% (95% confidence interval (CI) = 20.4-23.6; *I^2^* = 99.92%, *P* < 0.0001). The studies included in the meta-analysis were analyzed by subgroups. The pooled prevalence of stunting in children aged 2-5 years old and children aged 6 and older was 25.7% and 16.5%, respectively. The pooled prevalence of stunting was 34.1% in rural and 12.4% in urban areas. The pooled prevalence of stunting according to HDI was 30.1%, 28.5%, 13.1%, in low, medium, and high HDI countries, respectively. Furthermore, the pooled prevalence of stunting according to war status was 28.5% in war-involved countries vs 20.6% in others.

**Conclusions:**

High prevalence of malnutrition was seen based on stunting indicator in the meta-analysis study in the MENA region, and this issue became more pronounced when the data was divided into subgroups based on age, residential area, and HDI. Inequality regarding social, economic, and political factors leads to significant malnutrition in the mentioned region.

Malnutrition is a major public health problem, and world widely among children the three leading risk factors attributable to DALYs were all related to malnutrition [1]. In developing countries, it is an underlying factor in over 50% of the 10–11 million annual preventable deaths in children under 5 years of age [2]. Malnutrition is an extremely common disorder, associated with high rates of mortality and morbidity. It requires specialized treatment and prevention interventions [2]. Despite improvements in recent years, millions of children are deprived of their rights to optimal nutrition [3], and as a direct result of rights violations, children in the poorest households in low-income countries are twice as likely to die before the age of 5 years and twice as likely to be stunted because of chronic malnutrition compared to children in the richest households [3]. Stunting is one of the most common effects of chronic malnutrition in the world. The global percentage of stunted children under 5 years of age (CU5) was 21.3% based on UNICEF/WHO/World Bank Joint Child Malnutrition Estimates, March 2020 edition [4]; the prevalence of children's stunting was 24% in the 11 countries of the Middle East-North Africa (MENA) region based on a survey conducted during 2003–2016 [5], which seems to be higher than the mean world estimates.

Based on UNICEF’s conceptual framework [6], a child’s dietary intake and exposure to disease are affected by underlying factors, including household food insecurity (lack of availability, access to, and/or utilization of a diverse diet), inadequate care and feeding practices for children, unhealthy household and surrounding environments, and inaccessible and often inadequate health care, all of which could present inequity in a community or household. Basic causes of poor nutrition encompass the societal structures and processes that neglect human rights and perpetuate poverty, limiting or denying the access of vulnerable populations to essential resources. Social, economic, and political factors can have a long-term influence on maternal and childhood under-nutrition. Moreover, chronic malnutrition can lead to poverty, creating a vicious cycle [7].

The MENA region comprises 23 countries and territories, which has vast reserves comprising 60% of the world's petroleum and 45% of the world's natural gas reserves [8]. Due to these rich resources, combined with its location between three continents, (Asia, Africa, and Europe), the MENA region has been embroiled in conflict for a long time, with the highest peak occurring in the 21st century with the Iraq war, the rise of ISIS, the Syrian War, and the Yemeni war [9].

The Human Development Index (HDI) is a statistical composite index of life expectancy, education, and per capita income indicators used to rank countries into four tiers of human development. Studies have revealed an association between HDI index, malnutrition, and child mortality [10]. A country scores a higher HDI when the lifespan of its population, the education level, and the gross national income GNI (PPP) per capita are higher [10]. MENA countries categorized based on their HDI score are shown in Table S1 in the **Online Supplementary Document**.

Humanitarian crises, such as civil wars, conflicts, and disasters, have devastating impacts on health, especially in vulnerable groups such as children. This population group continues to be affected disproportionately by widespread conflict and often bears many more long-term consequences than adults [11]. The consequences of war and conflict occur in several ways, including death and injuries, adverse and long-term developmental consequences like chronic malnutrition and mental growth, and psychological disorders [11].

Given the strategic importance of the MENA region, the state of war and inequity in the region and its effect on malnutrition that leads to mortality and reduced economic development in this region, the current study aimed to examine the prevalence of stunting as an indicator of chronic malnutrition in the MENA region in children and adolescents, considering age, HDI, living area and war involved countries as an indicator for inequity.

## METHODS AND MATERIALS

### Search strategy

The electronic databases of PubMed, SCOPUS, Web of Science, and Embase were systematically searched. Given that local data for all the MENA countries was not available, only English keywords were searched. Keywords were obtained from MeSH and also extracted from related articles. The study syntax was formed from three components combined with the “AND” operator. The first component referred to all MENA countries searched together by the “OR” operator. Almost all countries in different definitions of the MENA region (ie, WHO and UNICEF) were included. The second component included words related to “child”, and “malnutrition”, and child growth-related expressions form the third component. In the last decade, Changes and wars have taken place in the MENA region. Yemen insurgency happened in 2009 and MENA experienced Arab spring in 2010 which seems to be a beginning for new evolutions in this region. Therefore, the articles that were published between January 1, 2009 and December 31, 2019 were included. The last search was done in September 2020. Our search strategy is shown in Table S2 in the **Online Supplementary Document**. Analyses were performed in accordance with the guidelines proposed by PRISMA.

### Study selection

The authors independently conducted the search phase and screening stage (KP), selection (KP and A.M.), quality assessment (JH, KP, and AS), and data extraction (AM and AS). Any disagreement was resolved by consensus, and if the disagreement was not resolved, a third expert opinion was sought.

The POLIS (population, outcome, location, indicator, study design) criteria used to perform the systematic review are outlined in Table S3 in the **Online Supplementary Document****.**

### Inclusion and exclusion criteria

**Figure 1:**
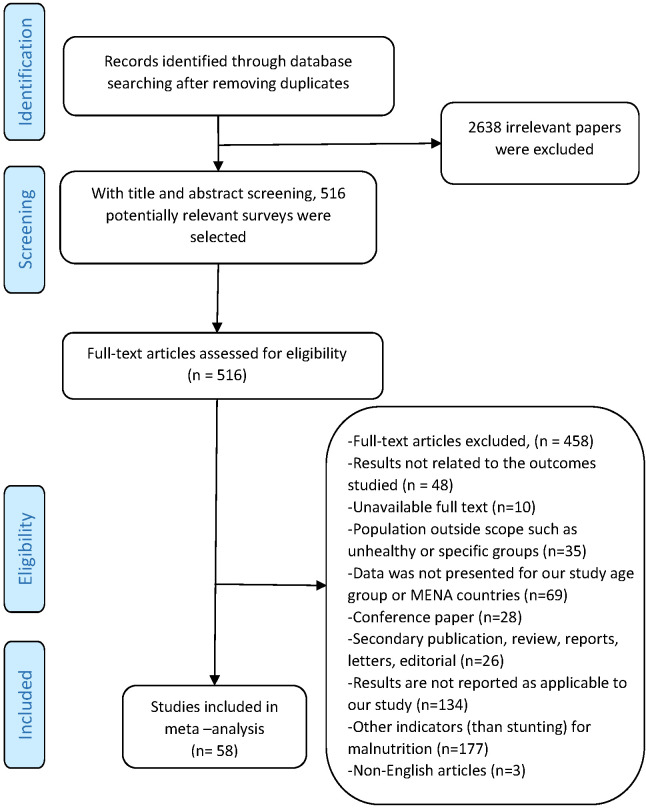
Follow diagram of systematic review and searches for stunting among children 2-18 years old in the Middle East and North Africa (MENA) region.

Cross-sectional and cohort studies conducted on children who were living in MENA countries including refugees were included in the study. Children of all ages were included in the search but for final inclusion, studies conducted on children 2 to 18 years old were selected. Studies on malnourished children, specific groups such as working children, and children referred to a hospital or clinic for malnutrition or other illnesses were excluded. Qualitative studies, commentaries, letters, and editorials were also excluded as well as conference abstracts, articles without the full text, and non-English reports and papers. **Figure 1** shows the process to exclude the unrelated articles.

### Definition of stunting

Based on the WHO definition, stunting is the impaired growth and development that children experience from poor nutrition, repeated infection, and inadequate psychosocial stimulation. Children are defined as stunted if their height-for-age was more than two standard deviations below the median of the WHO Child Growth Standards [12].

### Article screening and data extraction

In the primary screening, two authors (A.M. and K.A.) independently reviewed the title and abstract of studies collected from the search phase based on the inclusion and exclusion criteria. The studies that failed to pass the eligibility criteria were excluded at this stage. In the secondary screening, the full texts of these articles were reviewed with regard to the inclusion criteria. In the case of missing data or unavailable full texts in the included studies, attempts were made to access the authors’ contact data through two emails over a one–month period. No response to the email resulted in the removal of the article. Then the data of each of the included studies was extracted, and it is briefly presented in **Table 1**.

**Table 1:** Characteristics of the included studies in the systematic review and meta-analysis

No.	Author/year	Year of data collection	Country	Urban/rural/refugee camp	Sample size	Study design	Age (range)	Sex
1	Kia (2019) [13]	2010	Iran	urban/rural	30 960	cross-sectional	<5	M/F
2	Campisi (2019) [14]	2006- 2010	Pakistan	urban	1872	cross- sectional	<5	
3	Pernitez-Agan (2019) [15]	2015- 2016	Syria	refugee	14 552	cross-sectional	5-59 m	M/F
4	Engidaw (2019) [16]	2015	Ethiopia	refugee	423	cross-sectional	10-19 y	F
5	Farooq U.(2019) [17]	2012-2013	Pakistan	urban/rural	3184	cross-sectional	7-14 y	M/F
6	Pradeilles (2019) [18]	2015-2017	Pakistan	rural	1161	longitudinal	9-15 m	M/F
7	Khan (2019) [19]	2012-2013	Pakistan	urban/rural	3071	cross-sectional	<5	M/F
8	Kishk (2019) [20]	2017	Palestine	refugee	2399	cross-sectional	grade 1	M/F
9	Al Maghaireh (2019) [21]	NM	Jordan	not mentioned	453	cross-sectional	6-12 y	M/F
10	Walpole (2018) [22]	2016	Northern Greece	refugee camp	177	Cross-sectional	<5	M/F
11	Sharaf (2018) [23]	2014	Egypt	urban/rural	13 682		0-4 y	M/F
12	Sassi (2018) [24]	2009-2010	North Africa	urban	437	Cross-sectional	6-59 m	M/F
13	Rashad (2019) [25]	2008	Egypt	urban/rural	45 600	repeated cross-sectional data	<5	M/F
14	Kapoor (2018) [26]	2016	Pakistan	urban	300		12-17 y	M/F
15	Rashad (2019) [25]	2014	Egypt	urban/rural	12 888	Cross-sectional	<5	M/F
16	Style (2017) [27]	2008-2011	Horn of Africa	refugee camp	2500	Cross-sectional	6-59 m	M/F
17	Almasian (2017) [28]	2010	Iran	urban/rural	8443	Cross-sectional	<5	M/F
18	Jawad (2017) [29]	2016	Iraq	urban/rural	1000	Cross-sectional	<5	M/F
19	Kinyoki (2017) [30]	2007-2010	Somalia	urban, rural	73 778	several cross- sectional surveys	6-59 m	M/F
20	Zainab (2016) [31]	2016	Pakistan	urban/rural	385	Cross-sectional	10-14 y	M/F
21	Veghari (2016) [32]	1998-2013	Iran	Rural	7575	Cross-sectional	<5	M/F
22	Shahraki (2016) [33]	2013-2014	Iran	urban/rural	610	Cross-sectional	7-11 y	M/F
23	Massad (2016) [34]	2015	Palestine	Palestine Refugees	1484	cross-sectional	5-16 y	M/F
24	Khatibi (2016) [35]	NM	Iran	urban	443	cross-sectional	2-6 y	M/F
25	Veghari (2015) [36]	2013	Iran	Urban	2530	cross-sectional	<5	M/F
26	Nuruddin (2015) [37]	1992-1993	Pakistan	Rural	1051	Cross-sectional	0-35 m	M/F
27	Khan (2015) [38]	NM	Pakistan	urban, rural	684	Cross-sectional	5-14 y	M/F
28	Mohamed (2015) [39]	2014	Sudan	Rural	835	Cross-sectional	6-14 y	
29	Kinyoki (2015) [40]	2007-2010	Somalia	urban, rural	1066	Cross-sectional	6-59 m	M/F
30	Motbainor (2015) [41]	2013	Ethiopia	urban/rural	3964	cross-sectional	<5	M/F
31	Turab (2014) [42]	2003, 2007 2012	Pakistan	Rural	265	cohort	<5	
32	Psaki (2014) [43]	2009-2010	Pakistan	rural	98	Cross-sectional	24-60 m	M/F
33	Nouri (2014) [44]	2011	Iran	urban/rural	902	Cross-sectional	<5	M/F
34	Musa (2014) [45]	2011	Sudan	urban/rural	505	Cross-sectional	<5	
35	Kelishadi (2014) [46]	2009	Iran	urban/rural	955 388	Cross-sectional	6 y	M/F
36	Kavosi (2014) [47]	2012-2013	Iran	urban/rural	15 278	Cross-sectional	<5	M/F
37	Nouri (2014) [48]	2011	Iran	urban/rural	2525	cross-sectional	<5	
38	Egypt survey (2014) [49]	2014	Egypt	urban/rural	7976	Cross-sectional	<5	M/F
39	UNICEF, Kabul report (2014) [50]	2013	Afghanistan	urban/rural	12 977	cross-sectional	<5	
40	Palestinian survey (2014) [51]	2014	Palestine		4163	cross-sectional	<5	M/F
41	Hioui (2013) [52]	NM	morocco		162	Cross-sectional	12-15 y	M/F
42	Shafieian (2013) [53]	NM	Iran	Urban	671	Cross-sectional	24-59 m	
43	Radi (2013) [54]	2009	Gaza	urban/rural/ refugee	733	Cross-sectional	2-5 y	M/F
44	Payandeh (2013) [55]	2004	Iran		70 339	Cross-sectional	<5	M/F
45	Pakistan survey (2013) [56]	2012-2013	Pakistan	urban/rural	2166	cross-sectional	<5	M/F
46	Nguyen (2013) [57]	2008-2014	Ethiopia		2962	Cross-sectional	<5	M/F
47	Mushtaq (2012) [58]	2009	Pakistan	urban/rural	1860	Population Based Study	5-12 y	M/F
48	Mansourian (2012) [59]	2009-2010	Iran	Urban/rural	5430	CASPIAN-III study	10-19, 15-19 y	
49	Veghari (2012) [60]	2010	Iran	urban/rural	5698	Cross-sectional		M/F
50	UNICEF, Afghanestan survey (2012) [61]	2010-11	Afghanistan	urban/rural	8566	cross-sectional	<5	M/F
51	Mohammadinia (2012) [62]	2010-2011	Iran	urban	700	cross-sectional	<5	M/F
52	Kanoa (2011) [63]	NM	Gaza strip	urban, refugee	571	Cross-sectional	5-6 y	M/F
53	Motlagh (2011) [64]	2008	Iran	urban/rural	862 433	cross-sectional		M/F
54	Mushtaq (2011) [58]	2009	Pakistan	urban/rural	1860	cross-sectional	5-12 y	M/F
55	Khatib (2010) [65]	2004	Jordan	refugee	325	Cross-sectional	6 m-10 y	M/F
56	Mulugeta (2010) [66]	2004-2005	Ethiopia	rural	318	Cross-sectional	<5	M/F
57	Rizwana (2010) [67]	2009	Pakistan	urban	344	cross-sectional	5-10 y	M/F
58	Sharifzadeh (2010) [68]	2007	Iran	urban/rural	1807	cross-sectional	<5	M/F

y – year, m – month

### Quality assessment

Three reviewers (JH, KP and AS) independently assessed the quality of the included studies, and disagreements were resolved through consensus. The corresponding review author was also consulted whenever necessary. The Newcastle-Ottawa Scale checklist was used to assess the quality of observational studies. Articles were considered to be of high quality when the total score was ≥7, fair quality if the score was ≥5 and <7, and poor quality if the score was lower than 5 (Table S4 in the **Online Supplementary Document**) [69].

### Statistical analysis

All statistical analyses for evaluating the proportion/frequency of stunting were performed using STATA version 14 (Stata Corp, College Station, TX, USA). As it seemed that there was heterogeneity, the random effect model was used. To evaluate the heterogeneity between studies, the Q and I^2^ statistic tests were used. For the Q test, a *P* < 0.05 was considered as statistically significant and I^2^ values of 75%, 50%, and 25% were considered as evidence of high, moderate, and low levels of heterogeneity, respectively. Frequency/proportion of stunting with 95% confidence interval was calculated. Estimates were pooled using a random effect model (REM). Subgroup analyses were done based on age (2-5 years/6-18 years old), rural/urban area and refugees, HDI score (low, medium, high, very high) (categories shown in Table S1 in the **Online Supplementary Document**), and war-involved countries.

### Publication bias and sensitivity analysis

Egger and Funnel Plot tests were used to evaluate the publication bias in the results. Funnel plots related to the publication bias are shown in Figure S1 in the **Online Supplementary Document**. The sensitivity analysis was done based on the quality of the studies. As the pooled prevalence was not significantly different between groups (*P* = 0.99), all studies were included in the meta-analysis.

## RESULTS

This study was conducted to investigate the differences between child stunting as an important indicator of malnutrition in MENA region countries according to their socio-economic and political status and due to the strategic nature of the region which influences malnutrition and consequent future economic development of these countries. A total of 3154 studies were obtained from the literature search after duplicates were eliminated. Of the remaining studies, 2638 were excluded due to irrelevancy. Thus, 516 potentially relevant studies were selected and their full texts assessed for eligibility according to the inclusion and exclusion criteria based on the information provided in the title and the abstract. Another 458 articles were excluded for the following reasons: results not related to the outcomes studied (n = 48), text not available (n = 10), population outside scope (n = 35), secondary publications (n = 26), non-applicable report of the study (n = 134), other indices than stunting for malnutrition (n = 177), and conference paper (n = 28). Finally, as shown in **Figure 1**, 58 studies were included in the systematic review and meta-analysis. Based on the sensitivity analysis, there was no significant error in the results (*P* = 0.12).

### Characteristics of the Included Studies in the Systematic Review

Of the 58 included studies, 56 were cross-sectional and 2 were cohort studies [18,42]. The study population was 2- to 18-year-olds. The total number of participants in the studies ranged from 98 subjects to 955 388 subjects, with a total of 2 202 869 subjects included in the present systematic review. Table 1 shows the characteristics of the included studies in detail.

Of the 58 studies included in the systematic review, according to the Development Human Index, 24 (41.4%) were conducted in regions with very high development human index (Bahrain, Emirates, Kuwait, Oman, Qatar, Saudi Arabia) or high development human index (Egypt, Iran, Jordan, Lebanon, Libya, Tunisia, Algeria) [13,21,23,25,28,32,33,35,36,44,46-49,53,55,59,60,62,64,65,68], 21 (36.2%) were conducted in regions with a medium development human index (Iraq, Morocco, Pakistan, Palestine) [14,17-20,26,29,31,34,37,38,42,43,51,52,54,56,58,63,67,70], and 13 (22.4%) were conducted in regions with a low development human index (Afghanistan, Djibouti, Sudan, Syria, Yemen, Somalia) [15,16,22,27,30,39-41,45,50,57,61,66].

Of the 58 observational studies included in the systematic review, eight (13.8%) were conducted in countries involved in a civil war [15,29,30,39,40,45,50,61], 42 (72.4%) were reported from both urban and rural areas, 8 (13.8%) were conducted in urban areas only [14,24,26,35,36,53,62,67], and 8 (13.8%) in rural areas only [18,32,37,39,42,43,66,70]. Also, 7 (12.1%) studies involved refugees [15,16,20,22,27,34,65].

Of the 58 studies, 39 (67.2%%) [13,15,18,19,22-24,27-30,32,36,37,40-42,44,45,48-51,53-57,61,62,66] had data on stunting in children aged 2-5 years, and 19 (32.8%) on children aged 6 years and older [14,16,17,20,21,26,31,33,34,38,39,46,52,58,59,63,64,67,70].

### Meta-analysis

**Figure 2:**
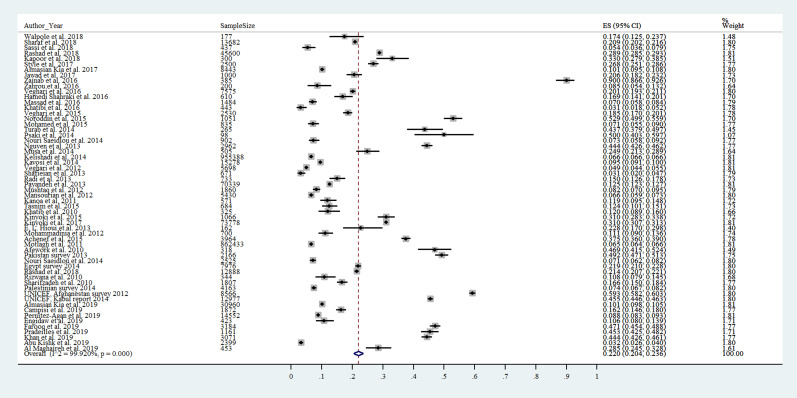
Pooled prevalence of stunting among children living in the Middle East and North Africa (MENA) region.

Based on the analysis of 58 studies (n = 2 202 869) evaluating variables at once, in general, the prevalence of stunting in children in the total MENA region was 22.0% (95% confidence interval (CI) = 20.4-23.6; *I^2^* = 99.92%, *P* < 0.0001) (**Figure 2**).

The 58 studies included in the meta-analysis were analyzed by subgroups depending on the age (2-5 years old, 6-18 years old), rural/urban area, HDI, and war-involved countries.

### Subgroup analyses

#### Age

A total of 33 studies were conducted in children aged 2-5 years old.

The pooled prevalence of stunting according to age group in descending order was as follows: children aged 2-5 years old (33 studies, *P* = 25.7%, 95% CI = 21.5-29.9; *I^2^* = 99.89%, *P* < 0.001) and children aged 6 years and older (19 studies, *P* = 16.5%, 95% CI = 15.5-17.5; *I^2^* = 99.68%, *P* < 0.001). The heterogeneity was significantly different between groups (*P* < 0.001) (Figure S2 in the **Online Supplementary Document**).

#### Rural/urban area and refugees

The pooled prevalence of stunting according to rural/urban area and refugees in descending order was as follows: rural (8 studies, *P* = 34.1%, 95% CI = 22.4-45.8; *I^2^* = 99.4%, *P* < 0.001), urban (8 studies, *P* = 12.4%, 95% CI = 7.0-17.8; *I^2^* = 98.42%, *P* < 0.001), and refugees (7 studies, *P* = 12.1%, 95% CI = 7.4-16.8; *I^2^* = 99.36%, *P* < 0.001). The heterogeneity was significantly different between groups (*P* < 0.01) (Figure S3 in the **Online Supplementary Document**).

#### HDI

The pooled prevalence of stunting according to HDI in descending order was as follows: low HDI countries (13 studies, *P* = 30.1%, 95% CI = 20.8-39.4; *I^2^* = 99.91%, *P* < 0.001), medium HDI countries (21 studies, *P* = 28.5%, 95% CI = 20.2-36.7, *I^2^* = 99.78%, *P* < 0.001), and high HDI countries (23 studies, *P* = 13.1%, 95% CI = 11.7-14.4; *I^2^* = 99.89%, *P* < 0.001). The heterogeneity was significantly different between groups (*P* < 0.001) (Figure S4 in the **Online Supplementary Document**).

#### War-involved countries

The pooled prevalence of stunting according to war status in descending order was as follows: countries involved in war during 2009-2019 such as Syria, Yemen, Afghanistan, Iraq, Sudan, and Somalia (8 studies, *P* = 28.5%, 95% CI = 16.2-40.9; *I^2^* = 99.95%, *P* < 0.001) and countries not involved in war (50 studies, *P* = 20.6%, 95% CI = 19.3-21.8; *I^2^* = 99.86%, *P* < 0.001). The heterogeneity was not significantly different between groups (*P* = 0.208) (Figure S5 in the **Online Supplementary Document**).

### Countries

**Figure 3:**
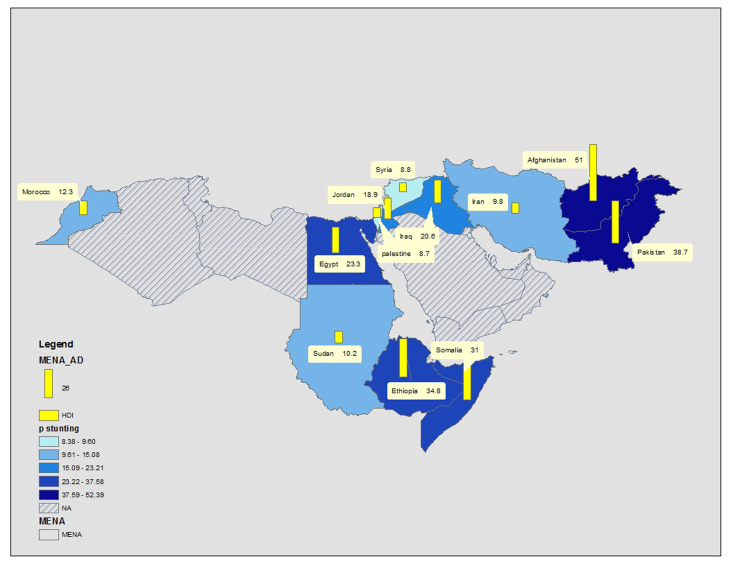
Stunting prevalence shown by percentage and by bar. The grey color shows the countries that have no databased on the inclusion criteria. More colorful countries have higher stunting prevalence.

The pooled prevalence of stunting according to countries in descending order was as follows: Afghanistan (2 studies, *P* = 51.0%, 95% CI = 50.4-51.7; *I^2^* and *P*-value not estimated), Pakistan (13 studies, *P* = 38.7%, 95% CI = 6.0-51.4; *I^2^* = 99.74%, *P* < 0.001), Ethiopia (4 studies, *P* = 34.8%, 95% CI = 21.6-48.0; *I^2^* = 99.23%, *P* < 0.001), Somalia (2 studies, *P* = 31.0%, 95% CI = 30.7-31.3; *I^2^* and *P*-value not estimated), Egypt (3 studies, *P* = 23.3%, 95% CI = 18.7-27.9; *I^2^* = 99.51%, *P* < 0.001), Jordan (2 studies, *P* = 18.9%, 95% CI = 16.2-21.6; *I^2^* and *P*-value not estimated), Morocco (2 studies, *P* = 12.5%, 95% CI = 9.0-15.6; *I^2^* and *P*-value not estimated), Sudan (2 studies, *P* = 10.2%, 95% CI = 8.6-11.8; *I^2^* and *P*-value not estimated), Iran (17 studies, *P* = 9.8%, 95% CI = 9.0-10.6; *I^2^* = 99.62%, *P* < 0.001), Palestine (5 studies, *P* = 8.7%, 95% CI = 5.5-11.8; *I^2^* = 97.13%, *P* < 0.001). The heterogeneity was significantly different between groups (*P* < 0.001). **Figure 3** shows the results.

## DISCUSSION

Having a productive and healthy life is an essential right of humankind without any discrimination. In this regard, children are a top priority due to their vulnerability to vast varieties of hazards [3]. Achieving this right depends on both supportive environmental context and available responsive and comprehensive health care [3]. Malnutrition and its consequences are major obstacles to having healthy children and can be avoidable [71]. Different grades of malnutrition can be seen among and between different regions recognized by the United Nations. The MENA region followed by South Asia and Africa have higher rates of stunted children [72]. The current study revealed inequality in chronic malnutrition in the MENA region not only between countries, but also within them. Noticeably, chronic malnutrition can cause stunting, which is a lifelong threat [73].

Sadly, MENA is the most unequal region both between and within countries in the world based on having or not having oil and distribution of resources and wealth among their populations [74]. The Middle East has witnessed many political conflicts during the four past decades, such as civil or international wars through which its economy has been destroyed and poverty and migration have prevailed. Moreover, climate change, drought, and floods in this area have destroyed agricultural lands, thereby aggravating food shortages. Both factors have resulted in unequal increases in food and nutrition insecurity, especially among children [75]. This divergence was seen in the current study between Afghanistan with 51.0% (95% CI = 50.4-51.7) prevalence of stunted children and Palestine with 8.7% (95% CI = 5.5-11.8) prevalence; however, the average rate was 22.0% (95% CI = 20.4-23.6) for this region.

During the past decade, Iraq, Syria, Afghanistan, Yemen, Libya, Somalia, and South Sudan have been involved in domestic or international wars [71,72]. As a result, millions of people have had to evacuate their homelands and live in poor conditions in other near or far countries as “refugees” [76]. Among these people, children are the most vulnerable group with less security in their feeding. Most studies on refugees were conducted in Syrian and African camps, and total prevalence of stunting was 12.1% (95% CI = 7.4-16.8) with the maximum prevalence of 26.84% in refugee camps in the Horn of Africa and Afghanistan 51.0% (95% CI = 50.4-51.7), which was involved in a civil war from 2001 to 2014 and then another ongoing war since 2015. Syria and Yemen have been engaged in wars since 2011 and 2015, respectively, few studies from these countries could be included in the current review based on the inclusion criteria and hence, the real situation of these countries is unlikely to be reflected in the results.

Different levels of development are another factor associated with unequal stunting among children living in the MENA region. Oil-rich countries, eg, Saudi Arabia, Kuwait, and Qatar, have fewer stunted children compared with population-rich countries, eg, Egypt, Algeria, and Turkey [74]. The average rate of stunting in children who live in countries with high HDI is 13.1% (95% CI = 11.7-14.4), while this rate is 30.1% (95% CI = 20.8-39.4) in countries with low HDI. In line with the current study, an ecologic study on the available global data showed a significant discrepancy in children’s stunting between different clusters of countries based on their HDI ranking [77]. A comparative analysis in Africa showed that 40%, 26%, and 19% of the CU5 are stunted in Zambia, Kenya, and Ghana, respectively. Noticeably, stunting rates in male children and those living in deprived households are higher than those in girls and well-off families, and this inequality has been amplified over time [78]. Based on an ecological study on global data obtained from the WHO, a negative statistical correlation is seen between HDI and its components and prevalence of stunting in CU5 [77].

Residency area, rural or urban, is another contributing factor to the prevalence of stunting among CU5. The current systematic review revealed a higher prevalence of stunting in CU5 in rural areas than urban areas in countries within MENA. However, in India, the concentration of stunting in children who live in urban areas is higher than that found in rural areas. Access to food, safe water, and sanitation are likely determinants for such discrepancies [79].

### Limitations

Due to the fact that meta-analysis studies use data from other studies, there are always some limitations. In the present study, one of the main limitations was the lack of separated reports of the prevalence of malnutrition in girls and boys. Another limitation of this study was the lack of reporting on the prevalence of malnutrition as a percentage in some studies and reporting it with a Z score or mean which could not be combined with the current results. Furthermore, some studies on malnutrition did not meet the inclusion criteria. Therefore, studies conducted in Djibouti, Yemen, Lebanon, Libya, Tunisia, Algeria, Bahrain, Emirates, Oman, Qatar, and Saudi Arabia were not included in the present analysis.

Furthermore, studies published in local or non-English language journals and gray literatures were not included in the study due to lack of access which led to increased heterogeneity.

## CONCLUSION

A high prevalence of malnutrition was seen based on stunting indicators in the meta-analysis study in the MENA region, and this issue became more pronounced when the data was divided into subgroups based on age, residential area, and HDI. Inequality regarding social, economic, and political factors led to significant malnutrition in the mentioned region, and basic causes of poor nutrition including the societal structures that neglect human rights and prolong poverty as well as access to essential resources for vulnerable populations should be considered in policy-making.

## Additional material


Online Supplementary Document
