# Supplementary material for: The prevalence of stunting among children and adolescents living in the Middle East and North Africa region (MENA): A systematic review and meta-analysis
Source: J Glob Health. 2021 Dec 25;11:04070. doi: 10.7189/jogh.11.04070 (PMC8711751; doi:10.7189/jogh.11.04070)
Supplement: Online Supplementary Document [file jogh-11-04070-s001.pdf]

Supplement file

Table S1. MENA region countries categorized based on 2019 HDI

Low HDI <0.550	Medium HDI 0.550-0.699	High HDI 0.700-0.799	Very high HDI >0.800
Afghanistan	Iraq	Egypt	Bahrain
Djibouti	Morocco	Iran	Emirate
Syria	Pakistan	Jordan	Kuwait
Sudan	Palestine	Lebanon	Oman
Yemen		Libya	Qatar
Somalia (2017)		Tunisia	Saudi Arabia
Ethiopia		Algeria	

Supplemental file

Table S2- search strategy

(("Adolescent" OR “Teen” OR “Teenager” OR “Youth” OR "Child") AND (“Afghanistan” OR “Pakistan” OR “Algeria” OR “Djibouti” OR “Egypt” OR “Iran” OR “Morocco” OR “Somalia” OR “Sudan” OR “Tunisia” OR “Yemen” OR “Bahrain” OR “Iraq” OR “Jordan” OR “Kuwait” OR “Lebanon” OR “Libya” OR “Oman” OR “Palestine” OR “Qatar” OR "Saudi Arabia" OR “Syria” OR "United Arab Emirates" OR "MENA region" OR "Middle east and north of Africa") AND ("Child Nutrition Disorder" OR “Nutritional Deficiency” OR “Undernutrition” OR “Under nutrition” OR “Under-nutrition” OR “Malnourishment” OR "Malnutrition" OR "Growth Disorder" OR “Stunted” OR “Stunting” OR "Growth Chart" OR “Reference Growth Curve” OR "Height for age" OR "Height-for-age" OR "HAZ"))

Supplemental file

Table S3. POLIS (population, outcome, location, indicator, study design)

Topic	Prevalence of stunting in Middle East and North Africa region (MENA)
Population	2 to 18 years old children
Outcome	Malnutrition
Location	MENA
Indicator	Stunting (percent)
Study design	Cross-sectional, case-control, cohort

Supplement file:

Table S4. Methodological quality of included studies in review using new castle checklist.

No.	Author, year	selection				Comparability	Exposure			score	Quality
		1	2	3	4	1	1	2			
1	Mansourian, M.2012	*	*	*	*	*	*	*		7	High
2	Jawad, I. H. 2017	*	*	*	*	*	*	*		7	High
3	Al Maghaireh et al. 2019	*		*	*	*	*	*		6	Fair
4	Pradeilles et al. 2019		*	*	*	*	*			5	Fair
5	Kanoa, B. J. 2011		*	*	*	*	*	*		6	Fair
6	Rizwana et al. 2010	*	*	*	*	*	*	*		7	High
7	Nouri Saeidlou et al. 2014	*	*	*	*	**	*	*		8	High
8	E. L. Hioui et al. 2013			*	*	*	*	*		5	Fair
9	Khan, Tasnim 2015	*	*	*	*	**	*	*		8	High
10	Khatib, I. M. 2010		*	*	*	*	*			5	Fair
11	Kinyoki, D. K. 2015	*	*	*	*	*	*			6	Fair
12	Kinyoki, D. K. 2017	*	*	*	*	*	*	*		7	High
13	Mohammadinia, N 2012	*	*	*	*	**	*	*		8	High
14	Motbainor, Achenef 2015	*	*	*	*	**	*	*		8	High
15	Motlagh, M. E. 2011		*	*	*	*	*			6	Fair
16	Mulugeta, Afework 2010	*	*	*	*	*	*			6	Fair
17	Payande, A. 2013		*	*	*	**	*	*		7	High
18	Pakistan survey 2013	*	*	*	*	*	*	*		8	High
19	Egypt survey 2014		*	*	*	*	*	*		6	Fair

20	Palestinian survey 2014		*	*	*	**	*	*		7	High
21	UNICEF, Afghanistan survey 2012	*	*	*	*	**	*	*		8	High
22	UNICEF, Kabul report 2014	*	*	*	*	*	*			6	Fair
23	Rashad, A. S. 2018	*	*	*	*	**	*	*		8	High
24	Sharifzadeh, G. 2010	*	*	*	*	*	*	*		7	High
25	Almasian Kia A 2019		*	*	*	*	*	*		6	Fair
26	Campisi SC 2019		*	*	*	*	*	*		6	Fair
27	Kishk NA 2019	*	*	*	*	*	*	*		7	High
28	Khan S 2019		*	*	*	*	*	*		6	Fair
29	Farooq MU 2019	*	*	*	*	**	*	*		8	High
30	Engidaw MT 2019	*	*	*	*	**	*	*		8	High
31	Pernitez-Agan S 2019	*	*	*	*	**	*	*		8	High
32	S.C.Walpole 2018	*			**	*	**	*		7	High
33	Mesbah Fathi Sharaf 2018	*			**		**	*		6	Fair
34	Sonia Sassi 2018	*			**		**	*		6	Fair
35	Ahmed Shoukry Rashad 2018	*			**	*	**	*		7	High
36	Aneel Kapoor 2018				**		**	*		5	Fair
37	Abdollah Almasian Kia 2017	*			**		**	*		6	Fair
38	Saima Zainab 2016	*	*		**	*	**	*		8	High
39	Fatima Ezzahra Zahrou 2016 Rct trial	*	*		**	*	**	*		8	High
40	Gholamreza Veghari 2016	*	*	*	**		**	*		8	High
41	Soudabeh Hamedi Shahraki 2016	*		*	**	*	**	*		8	High
42	Salwa Massad 2016	*	*	*	**	*	**	*		9	High
43	Mozhgan Khatibi 2016	*			**		**	*		6	Fair

44	Gholamreza Veghari 2015	*		*	**	*	**	*		8	High
45	Rozina Noroddin 2015	*	*	*	**	*	**	*		9	High
46	Sarar Mohamed 2015	*		*	**		**	*		7	High
47	Stephanie R Psaki 2014	*	*		**	*	**	*		8	High
48	Sakineh Nouri Saeidlou 2014		*		**		**	*		6	Fair
49	Phuong Hong Nguyen 2013	*			**	*	**	*		7	High
50	Taha H. Musa 2014				**	*	**	*		6	Fair
51	Roya Kelishadi 2014	*	*		**		**	*		7	High
52	Sarah Style 2018	*	*		**		**	*		7	High
53	Ali Turab 2014	*			**	*	**	*		7	High
54	Elham Kavosi 2014	*	*	*	**	*	**	*		9	High
55	Gholamreza Veghari 2012	*	*		**	*	**	*		8	High
56	Tahereh Shafieian 2013	*			**	*	**	*		7	High
57	S.M. Radi 2013	*		*	**	*	**	*		8	High
58	Mushtaq, Muhammad Umair 2012	*	*		**	*	**	*		8	High

Figure S1

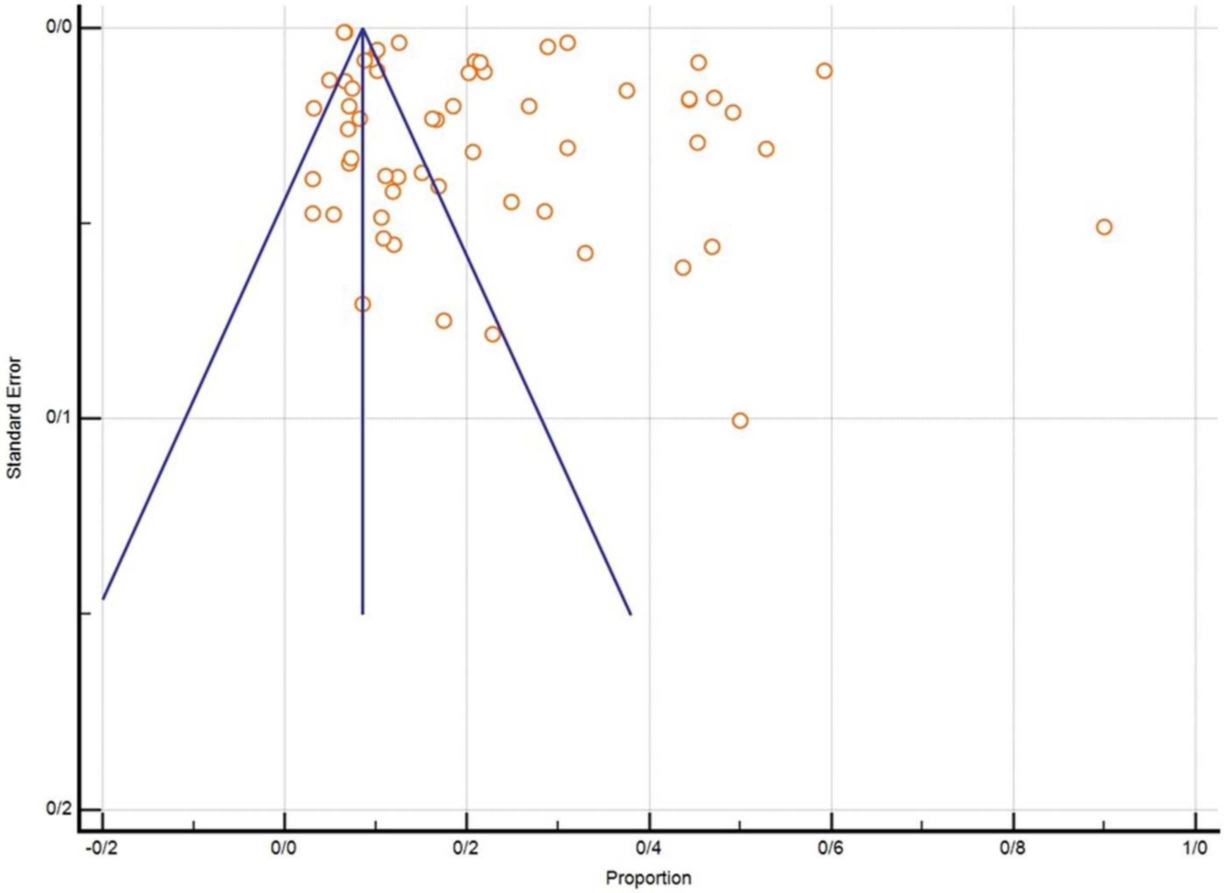

Figure S2

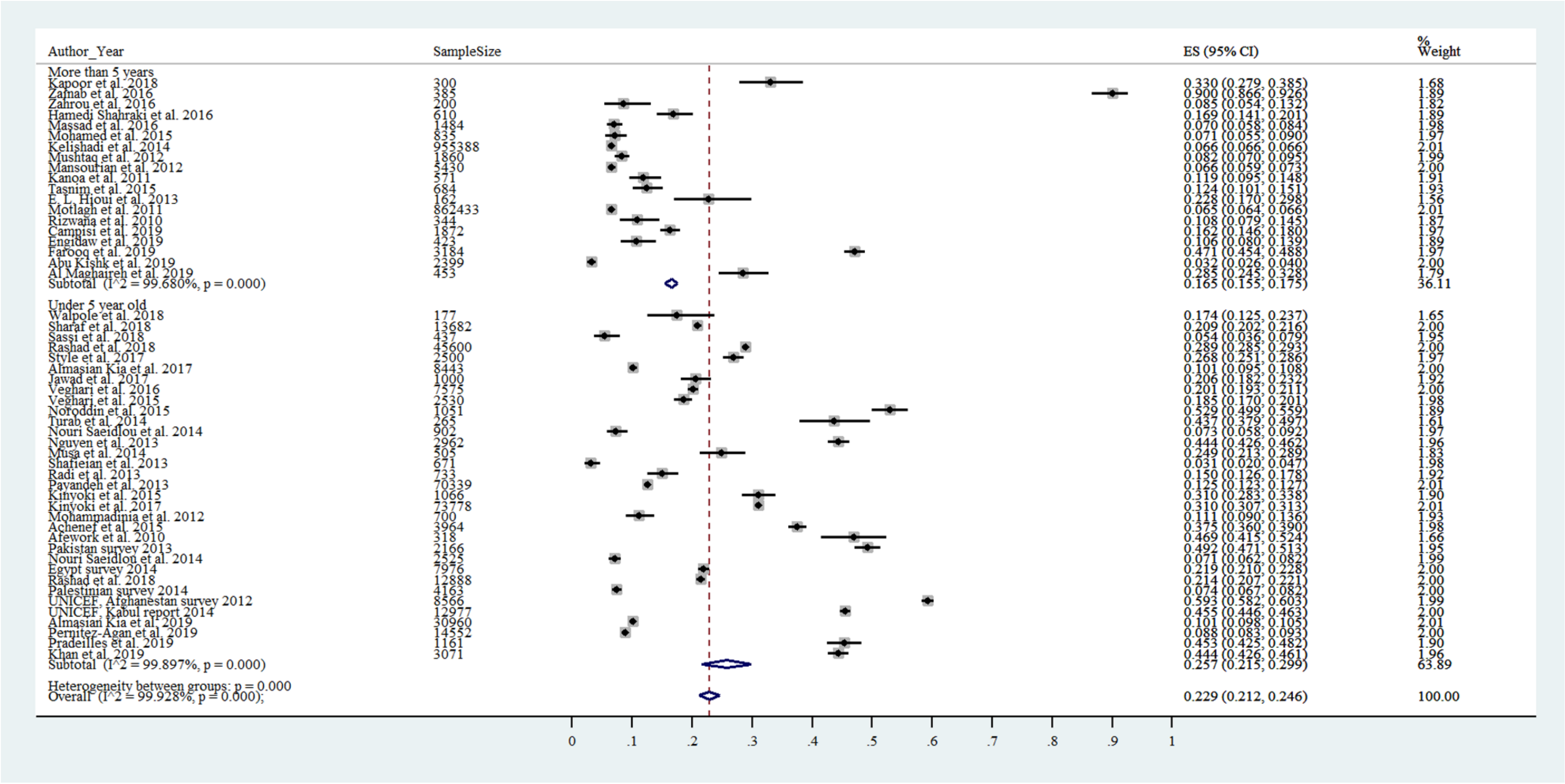
Author_Year SampleSize ES (95% CI) % Weight
More than 5 years
Kapoor et al. 2018 300 0.330 (0.279, 0.385) 1.68
Zainab et al. 2016 385 0.900 (0.866, 0.926) 1.89
Zahrou et al. 2016 200 0.085 (0.054, 0.132) 1.82
Hamedi Shahraki et al. 2016 610 0.169 (0.141, 0.201) 1.89
Massad et al. 2016 1484 0.070 (0.058, 0.084) 1.98
Mohamed et al. 2015 835 0.071 (0.055, 0.090) 1.97
Kelishadi et al. 2014 955388 0.066 (0.066, 0.066) 2.01
Mushtaq et al. 2012 1860 0.082 (0.070, 0.095) 1.99
Mansourian et al. 2012 5430 0.066 (0.059, 0.073) 2.00
Kanoa et al. 2011 571 0.119 (0.095, 0.148) 1.91
Tasnim et al. 2015 684 0.124 (0.101, 0.151) 1.93
E. L. Hioui et al. 2013 162 0.228 (0.170, 0.298) 1.56
Motlagh et al. 2011 862433 0.065 (0.064, 0.066) 2.01
Rizwana et al. 2010 344 0.108 (0.079, 0.145) 1.87
Campisi et al. 2019 1872 0.162 (0.146, 0.180) 1.97
Engidaw et al. 2019 423 0.106 (0.080, 0.139) 1.89
Farooq et al. 2019 3184 0.471 (0.454, 0.488) 1.97
Abu Kishk et al. 2019 2399 0.032 (0.026, 0.040) 2.00
Al Maghaireh et al. 2019 453 0.285 (0.245, 0.328) 1.79
Subtotal (I^2 = 99.680%, p = 0.000) 0.165 (0.155, 0.175) 36.11
Under 5 year old
Walpole et al. 2018 177 0.174 (0.125, 0.237) 1.65
Sharaf et al. 2018 13682 0.209 (0.202, 0.216) 2.00
Sassi et al. 2018 437 0.054 (0.036, 0.079) 1.95
Rashad et al. 2018 45600 0.289 (0.285, 0.293) 2.00
Style et al. 2017 2500 0.268 (0.251, 0.286) 1.97
Almasian Kia et al. 2017 8443 0.101 (0.095, 0.108) 2.00
Jawad et al. 2017 1000 0.206 (0.182, 0.232) 1.92
Veghari et al. 2016 7575 0.201 (0.193, 0.211) 2.00
Veghari et al. 2015 2530 0.185 (0.170, 0.201) 1.98
Noroddin et al. 2015 1051 0.529 (0.499, 0.559) 1.89
Turab et al. 2014 265 0.437 (0.379, 0.497) 1.61
Nouri Saeidlou et al. 2014 902 0.073 (0.058, 0.092) 1.97
Nguyen et al. 2013 2962 0.444 (0.426, 0.462) 1.96
Musa et al. 2014 505 0.249 (0.213, 0.289) 1.83
Shafieian et al. 2013 671 0.031 (0.020, 0.047) 1.98
Radi et al. 2013 733 0.150 (0.126, 0.178) 1.92
Payandeh et al. 2013 70339 0.125 (0.123, 0.127) 2.01
Kinyoki et al. 2015 1066 0.310 (0.283, 0.338) 1.90
Kinyoki et al. 2017 73778 0.310 (0.307, 0.313) 2.01
Mohammadinia et al. 2012 700 0.111 (0.090, 0.136) 1.93
Achenef et al. 2015 3964 0.375 (0.360, 0.390) 1.98
Afework et al. 2010 318 0.469 (0.415, 0.524) 1.66
Pakistan survey 2013 2166 0.492 (0.471, 0.513) 1.95
Nouri Saeidlou et al. 2014 2525 0.071 (0.062, 0.082) 1.99
Egypt survey 2014 7976 0.219 (0.210, 0.228) 2.00
Rashad et al. 2018 12888 0.214 (0.207, 0.221) 2.00
Palestinian survey 2014 4163 0.074 (0.067, 0.082) 2.00
UNICEF, Afghanestan survey 2012 8566 0.593 (0.582, 0.603) 1.99
UNICEF, Kabul report 2014 12977 0.455 (0.446, 0.463) 2.00
Almasian Kia et al. 2019 30960 0.101 (0.098, 0.105) 2.01
Pernitez-Agan et al. 2019 14552 0.088 (0.083, 0.093) 2.00
Pradeilles et al. 2019 1161 0.453 (0.425, 0.482) 1.90
Khan et al. 2019 3071 0.444 (0.426, 0.461) 1.96
Subtotal (I^2 = 99.897%, p = 0.000) 0.257 (0.215, 0.299) 63.89
Heterogeneity between groups: p = 0.000
Overall (I^2 = 99.928%, p = 0.000); 0.229 (0.212, 0.246) 100.00
0 .1 .2 .3 .4 .5 .6 .7 .8 .9 1

Figure S3

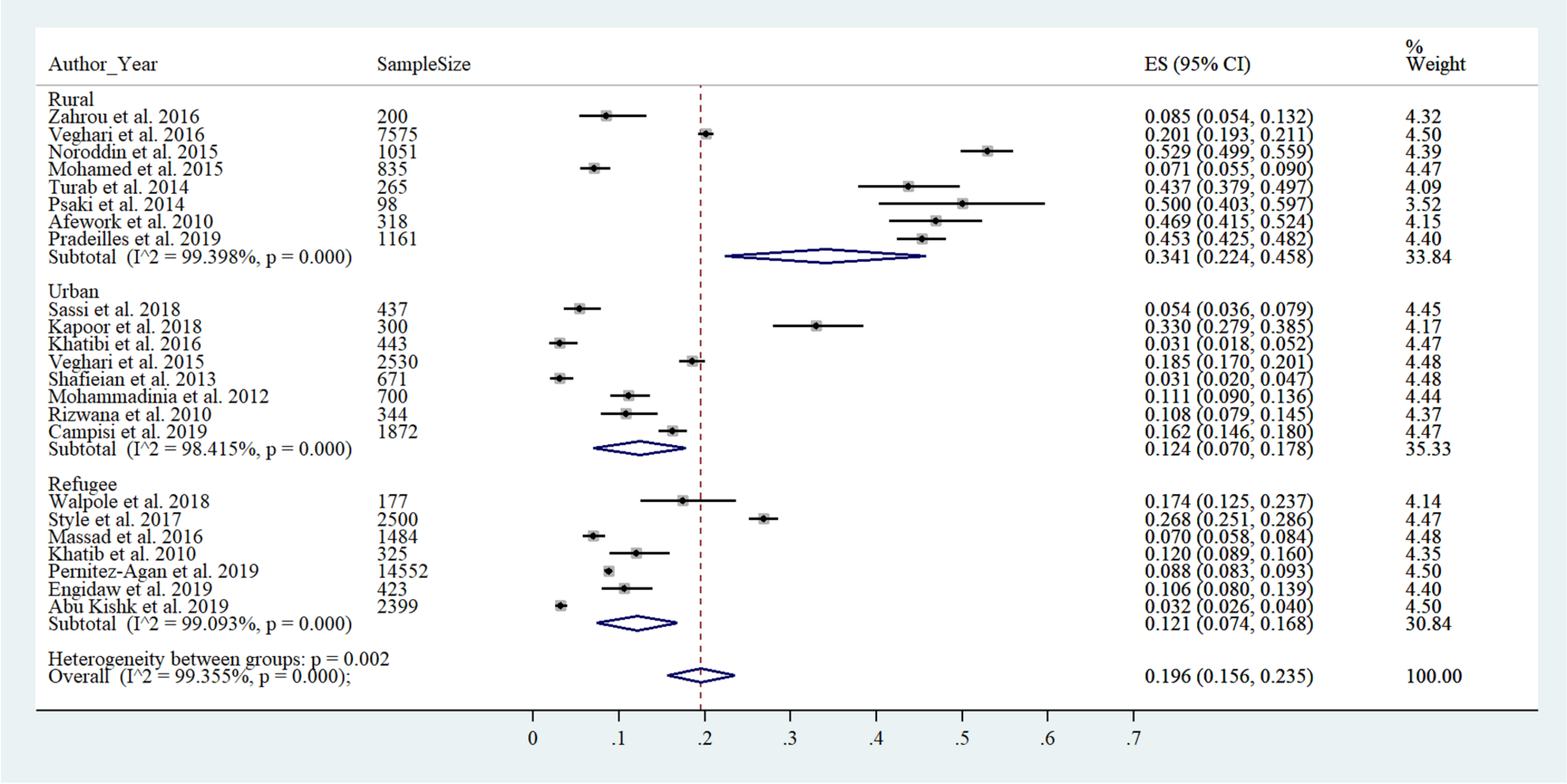

Author_Year
SampleSize
ES (95% CI)
% Weight
Rural
Zahrou et al. 2016 200 0.085 (0.054, 0.132) 4.32
Veghari et al. 2016 7575 0.201 (0.193, 0.211) 4.50
Noroddin et al. 2015 1051 0.529 (0.499, 0.559) 4.39
Mohamed et al. 2015 835 0.071 (0.055, 0.090) 4.47
Turab et al. 2014 265 0.437 (0.379, 0.497) 4.09
Psaki et al. 2014 98 0.500 (0.403, 0.597) 3.52
Afework et al. 2010 318 0.469 (0.415, 0.524) 4.15
Pradeilles et al. 2019 1161 0.453 (0.425, 0.482) 4.40
Subtotal (I^2 = 99.398%, p = 0.000) 0.341 (0.224, 0.458) 33.84
Urban
Sassi et al. 2018 437 0.054 (0.036, 0.079) 4.45
Kapoor et al. 2018 300 0.330 (0.279, 0.385) 4.17
Khatibi et al. 2016 443 0.031 (0.018, 0.052) 4.47
Veghari et al. 2015 2530 0.185 (0.170, 0.201) 4.48
Shafieian et al. 2013 671 0.031 (0.020, 0.047) 4.48
Mohammadinia et al. 2012 700 0.111 (0.090, 0.136) 4.44
Rizwana et al. 2010 344 0.108 (0.079, 0.145) 4.37
Campisi et al. 2019 1872 0.162 (0.146, 0.180) 4.47
Subtotal (I^2 = 98.415%, p = 0.000) 0.124 (0.070, 0.178) 35.33
Refugee
Walpole et al. 2018 177 0.174 (0.125, 0.237) 4.14
Style et al. 2017 2500 0.268 (0.251, 0.286) 4.47
Massad et al. 2016 1484 0.070 (0.058, 0.084) 4.48
Khatib et al. 2010 325 0.120 (0.089, 0.160) 4.35
Pernitez-Agan et al. 2019 14552 0.088 (0.083, 0.093) 4.50
Engidaw et al. 2019 423 0.106 (0.080, 0.139) 4.40
Abu Kishk et al. 2019 2399 0.032 (0.026, 0.040) 4.50
Subtotal (I^2 = 99.093%, p = 0.000) 0.121 (0.074, 0.168) 30.84
Heterogeneity between groups: p = 0.002
Overall (I^2 = 99.355%, p = 0.000); 0.196 (0.156, 0.235) 100.00
0
.1
.2
.3
.4
.5
.6
.7

Figure S4

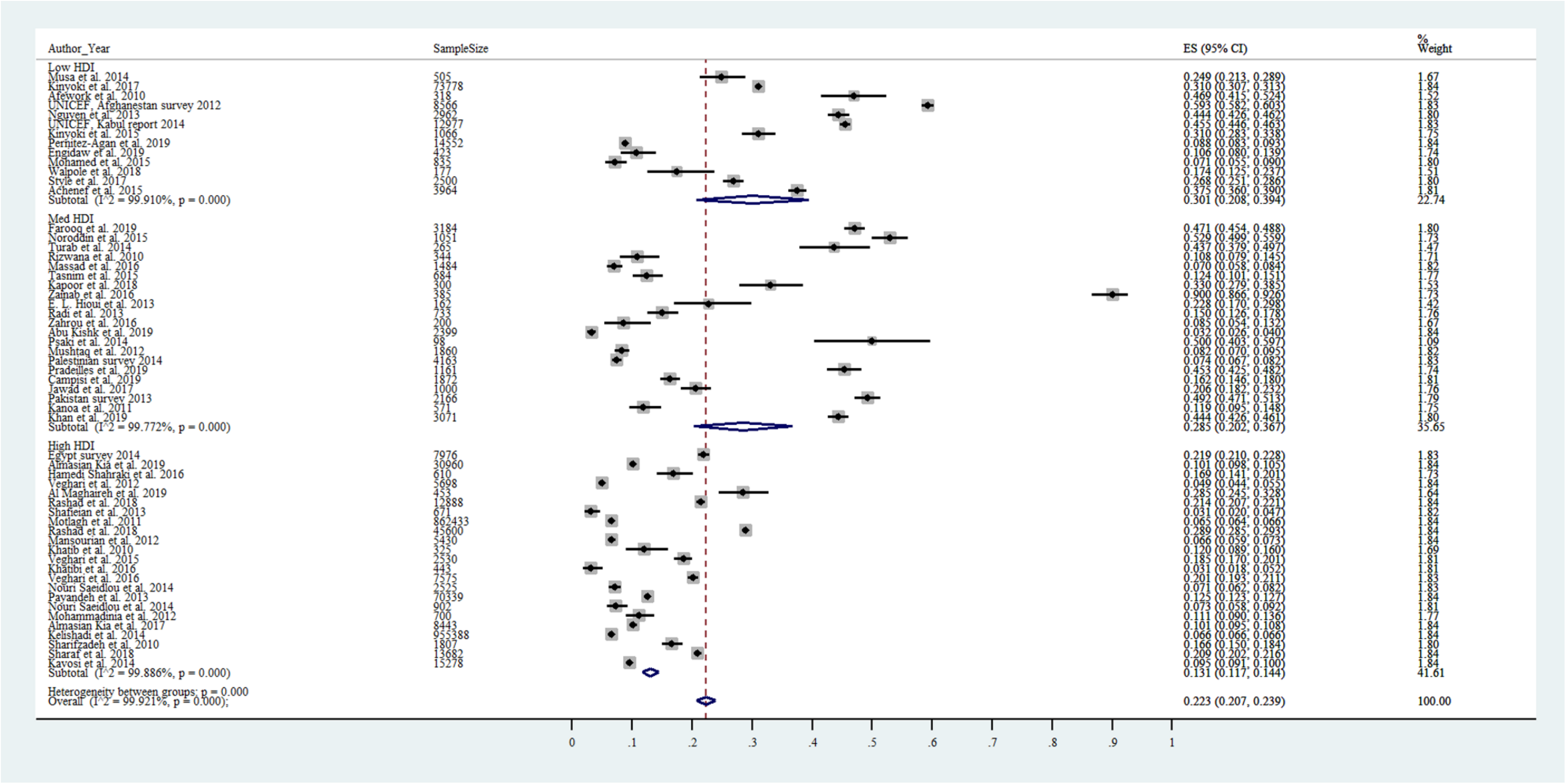

Author_Year SampleSize ES (95% CI) % Weight
Low HDI
Musa et al. 2014 505 0.249 (0.213, 0.289) 1.67
Kinyoki et al. 2017 73778 0.310 (0.307, 0.313) 1.84
Afework et al. 2010 318 0.469 (0.415, 0.524) 1.52
UNICEF, Afghanestan survey 2012 8566 0.593 (0.582, 0.603) 1.83
Nguyen et al. 2013 2962 0.444 (0.426, 0.462) 1.80
UNICEF, Kabul report 2014 12977 0.455 (0.446, 0.463) 1.83
Kinyoki et al. 2015 1066 0.310 (0.283, 0.338) 1.75
Pernitez-Agan et al. 2019 14552 0.088 (0.083, 0.093) 1.84
Engidaw et al. 2019 423 0.106 (0.080, 0.139) 1.74
Mohamed et al. 2015 835 0.071 (0.055, 0.090) 1.80
Walpole et al. 2018 177 0.174 (0.125, 0.237) 1.51
Style et al. 2017 2500 0.268 (0.251, 0.286) 1.80
Achenef et al. 2015 3964 0.375 (0.360, 0.390) 1.81
Subtotal (I^2 = 99.910%, p = 0.000) 0.301 (0.208, 0.394) 22.74
Med HDI
Farooq et al. 2019 3184 0.471 (0.454, 0.488) 1.80
Noroddin et al. 2015 1051 0.529 (0.499, 0.559) 1.73
Turab et al. 2014 265 0.437 (0.379, 0.497) 1.47
Rizwana et al. 2010 344 0.108 (0.079, 0.145) 1.71
Massad et al. 2016 1484 0.070 (0.058, 0.084) 1.82
Tasnim et al. 2015 684 0.124 (0.101, 0.151) 1.77
Kapoor et al. 2018 300 0.330 (0.279, 0.385) 1.53
Zainab et al. 2016 385 0.900 (0.866, 0.926) 1.73
E. L. Hioui et al. 2013 162 0.228 (0.170, 0.298) 1.42
Radi et al. 2013 733 0.150 (0.126, 0.178) 1.76
Zahrou et al. 2016 200 0.085 (0.054, 0.132) 1.67
Abu Kishk et al. 2019 2399 0.032 (0.026, 0.040) 1.84
Psaki et al. 2014 98 0.500 (0.403, 0.597) 1.09
Mushtaq et al. 2012 1860 0.082 (0.070, 0.095) 1.82
Palestinian survey 2014 4163 0.074 (0.067, 0.082) 1.83
Pradeilles et al. 2019 1161 0.453 (0.425, 0.482) 1.74
Campisi et al. 2019 1872 0.162 (0.146, 0.180) 1.81
Jawad et al. 2017 1000 0.206 (0.182, 0.232) 1.76
Pakistan survey 2013 2166 0.492 (0.471, 0.513) 1.79
Kanoa et al. 2011 571 0.119 (0.095, 0.148) 1.75
Khan et al. 2019 3071 0.444 (0.426, 0.461) 1.80
Subtotal (I^2 = 99.772%, p = 0.000) 0.285 (0.202, 0.367) 35.65
High HDI
Egypt survey 2014 7976 0.219 (0.210, 0.228) 1.83
Almasian Kia et al. 2019 30960 0.101 (0.098, 0.105) 1.84
Hamedi Shahraki et al. 2016 610 0.169 (0.141, 0.201) 1.73
Veghari et al. 2012 5698 0.049 (0.044, 0.055) 1.84
Al Maghaireh et al. 2019 453 0.285 (0.245, 0.328) 1.64
Rashad et al. 2018 12888 0.214 (0.207, 0.221) 1.84
Shafieian et al. 2013 671 0.031 (0.020, 0.047) 1.82
Motlagh et al. 2011 862433 0.065 (0.064, 0.066) 1.84
Rashad et al. 2018 45600 0.289 (0.285, 0.293) 1.84
Mansourian et al. 2012 5430 0.066 (0.059, 0.073) 1.84
Khatib et al. 2010 325 0.120 (0.089, 0.160) 1.69
Veghari et al. 2015 2530 0.185 (0.170, 0.201) 1.81
Khatibi et al. 2016 443 0.031 (0.018, 0.052) 1.81
Veghari et al. 2016 7575 0.201 (0.193, 0.211) 1.83
Nouri Saeidlou et al. 2014 2525 0.071 (0.062, 0.082) 1.83
Payandeh et al. 2013 70339 0.125 (0.123, 0.127) 1.84
Nouri Saeidlou et al. 2014 902 0.073 (0.058, 0.092) 1.81
Mohammadinia et al. 2012 700 0.111 (0.090, 0.136) 1.77
Almasian Kia et al. 2017 8443 0.101 (0.095, 0.108) 1.84
Kelishadi et al. 2014 955388 0.066 (0.066, 0.066) 1.84
Sharifzadeh et al. 2010 1807 0.166 (0.150, 0.184) 1.80
Sharaf et al. 2018 13682 0.209 (0.202, 0.216) 1.84
Kavosi et al. 2014 15278 0.095 (0.091, 0.100) 1.84
Subtotal (I^2 = 99.886%, p = 0.000) 0.131 (0.117, 0.144) 41.61
Heterogeneity between groups: p = 0.000
Overall (I^2 = 99.921%, p = 0.000); 0.223 (0.207, 0.239) 100.00
0
.1
.2
.3
.4
.5
.6
.7
.8
.9
1

Figure S5

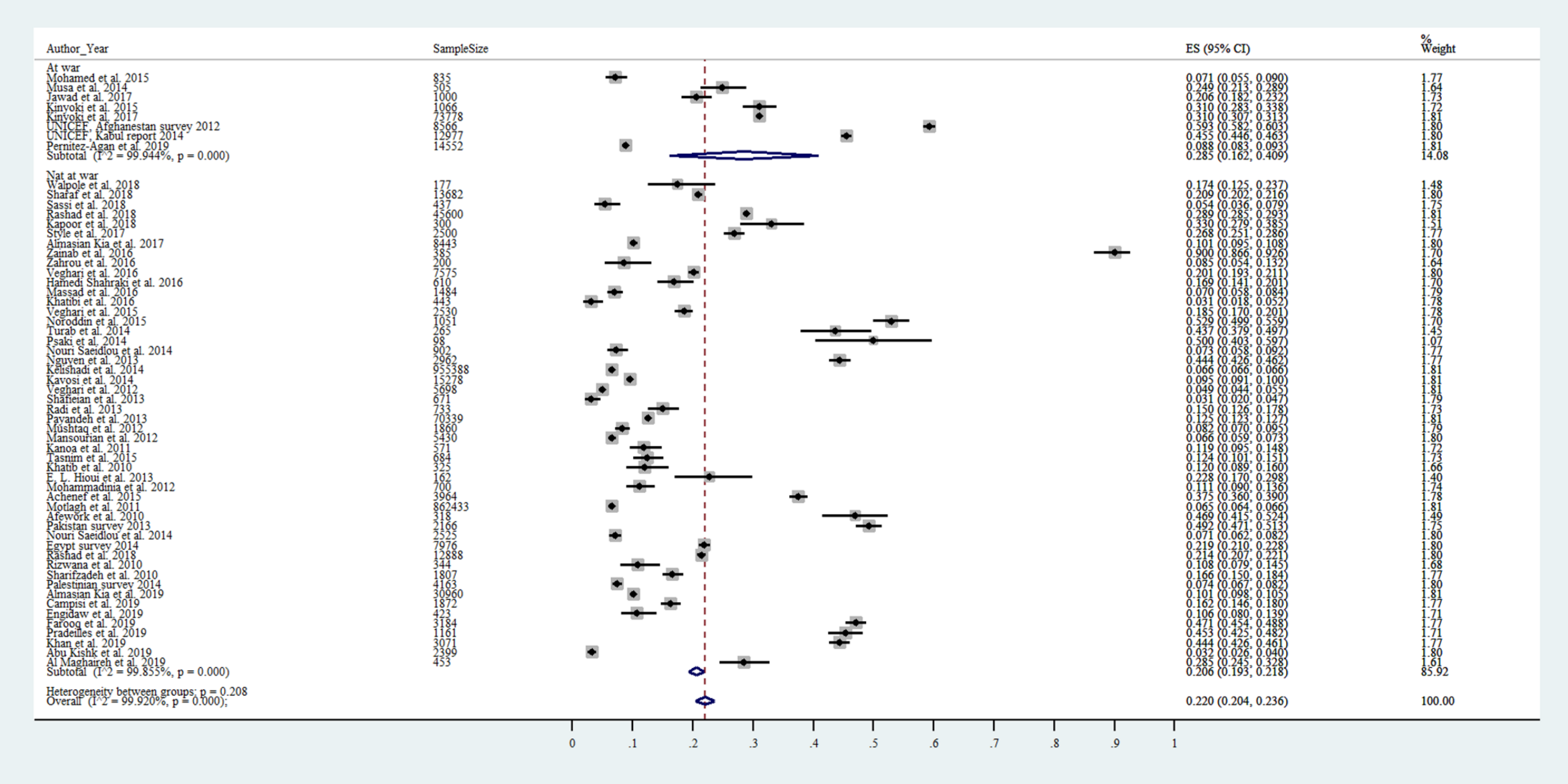
Author_Year SampleSize ES (95% CI) % Weight
At war
Mohamed et al. 2015 835 0.071 (0.055, 0.090) 1.77
Musa et al. 2014 505 0.249 (0.213, 0.289) 1.64
Jawad et al. 2017 1000 0.206 (0.182, 0.232) 1.73
Kinyoki et al. 2015 1066 0.310 (0.283, 0.338) 1.72
Kinyoki et al. 2017 73778 0.310 (0.307, 0.313) 1.81
UNICEF, Afghanestan survey 2012 8566 0.593 (0.582, 0.603) 1.80
UNICEF, Kabul report 2014 12977 0.455 (0.446, 0.463) 1.80
Pernitez-Agan et al. 2019 14552 0.088 (0.083, 0.093) 1.81
Subtotal (I^2 = 99.944%, p = 0.000) 0.285 (0.162, 0.409) 14.08
Nat at war
Walpole et al. 2018 177 0.174 (0.125, 0.237) 1.48
Sharaf et al. 2018 13682 0.209 (0.202, 0.216) 1.80
Sassi et al. 2018 437 0.054 (0.036, 0.079) 1.75
Rashad et al. 2018 45600 0.289 (0.285, 0.293) 1.81
Kapoor et al. 2018 300 0.330 (0.279, 0.385) 1.51
Style et al. 2017 2500 0.268 (0.251, 0.286) 1.77
Almasian Kia et al. 2017 8443 0.101 (0.095, 0.108) 1.80
Zainab et al. 2016 385 0.900 (0.866, 0.926) 1.70
Zahrou et al. 2016 200 0.085 (0.054, 0.132) 1.64
Veghari et al. 2016 7575 0.201 (0.193, 0.211) 1.80
Hamedi Shahraki et al. 2016 610 0.169 (0.141, 0.201) 1.70
Massad et al. 2016 1484 0.070 (0.058, 0.084) 1.79
Khatibi et al. 2016 443 0.031 (0.018, 0.052) 1.78
Veghari et al. 2015 2530 0.185 (0.170, 0.201) 1.78
Noroddin et al. 2015 1051 0.529 (0.499, 0.559) 1.70
Turab et al. 2014 265 0.437 (0.379, 0.497) 1.45
Psaki et al. 2014 98 0.500 (0.403, 0.597) 1.07
Nouri Saeidlou et al. 2014 902 0.073 (0.058, 0.092) 1.77
Nguyen et al. 2013 2962 0.444 (0.426, 0.462) 1.77
Kelishadi et al. 2014 955388 0.066 (0.066, 0.066) 1.81
Kavosi et al. 2014 15278 0.095 (0.091, 0.100) 1.81
Veghari et al. 2012 5698 0.049 (0.044, 0.055) 1.81
Shafieian et al. 2013 671 0.031 (0.020, 0.047) 1.79
Radi et al. 2013 733 0.150 (0.126, 0.178) 1.73
Payandeh et al. 2013 70339 0.125 (0.123, 0.127) 1.81
Mushtaq et al. 2012 1860 0.082 (0.070, 0.095) 1.79
Mansourian et al. 2012 5430 0.066 (0.059, 0.073) 1.80
Kanoa et al. 2011 571 0.119 (0.095, 0.148) 1.72
Tasnim et al. 2015 684 0.124 (0.101, 0.151) 1.73
Khatib et al. 2010 325 0.120 (0.089, 0.160) 1.66
E. L. Hioui et al. 2013 162 0.228 (0.170, 0.298) 1.40
Mohammadinia et al. 2012 700 0.111 (0.090, 0.136) 1.74
Achenef et al. 2015 3964 0.375 (0.360, 0.390) 1.78
Motlagh et al. 2011 862433 0.065 (0.064, 0.066) 1.81
Afework et al. 2010 318 0.469 (0.415, 0.524) 1.49
Pakistan survey 2013 2166 0.492 (0.471, 0.513) 1.75
Nouri Saeidlou et al. 2014 2525 0.071 (0.062, 0.082) 1.80
Egypt survey 2014 7976 0.219 (0.210, 0.228) 1.80
Rashad et al. 2018 12888 0.214 (0.207, 0.221) 1.80
Rizwana et al. 2010 344 0.108 (0.079, 0.145) 1.68
Sharifzadeh et al. 2010 1807 0.166 (0.150, 0.184) 1.77
Palestinian survey 2014 4163 0.074 (0.067, 0.082) 1.80
Almasian Kia et al. 2019 30960 0.101 (0.098, 0.105) 1.81
Campisi et al. 2019 1872 0.162 (0.146, 0.180) 1.77
Engidaw et al. 2019 423 0.106 (0.080, 0.139) 1.71
Farooq et al. 2019 3184 0.471 (0.454, 0.488) 1.77
Pradeilles et al. 2019 1161 0.453 (0.425, 0.482) 1.71
Khan et al. 2019 3071 0.444 (0.426, 0.461) 1.77
Abu Kishk et al. 2019 2399 0.032 (0.026, 0.040) 1.80
Al Maghaireh et al. 2019 453 0.285 (0.245, 0.328) 1.61
Subtotal (I^2 = 99.855%, p = 0.000) 0.206 (0.193, 0.218) 85.92
Heterogeneity between groups: p = 0.208
Overall (I^2 = 99.920%, p = 0.000); 0.220 (0.204, 0.236) 100.00
0 .1 .2 .3 .4 .5 .6 .7 .8 .9 1
